# Adsorption and Desorption Performance and Mechanism of Tetracycline Hydrochloride by Activated Carbon-Based Adsorbents Derived from Sugar Cane Bagasse Activated with ZnCl_2_

**DOI:** 10.3390/molecules24244534

**Published:** 2019-12-11

**Authors:** Yixin Cai, Liming Liu, Huafeng Tian, Zhennai Yang, Xiaogang Luo

**Affiliations:** 1 Beijing Advanced Innovation Center for Food Nutrition and Human Health, Beijing Technology & Business University (BTBU), Beijing 100048, China; yxcai0507@outlook.com (Y.C.);; 2 School of Chemical Engineering and Pharmacy, Wuhan Institute of Technology, LiuFang Campus, No.206, Guanggu 1st road, Donghu New & High Technology Development Zone, Wuhan 430205, Hubei Province, China; 3 School of Materials Science and Engineering, Zhengzhou University, No.100 Science Avenue, Zhengzhou 450001, Henan Province, China

**Keywords:** adsorption, desorption, mechanism, sugar cane bagasse, activated carbons, tetracycline

## Abstract

Adsorption and desorption behaviors of tetracycline hydrochloride by activated carbon-based adsorbents derived from sugar cane bagasse modified with ZnCl_2_ were investigated. The activated carbon was tested by SEM, EDX, BET, XRD, FTIR, and XPS. This activated carbon exhibited a high BET surface area of 831 m^2^ g^−1^ with the average pore diameter and pore volume reaching 2.52 nm and 0.45 m^3^ g^−1^, respectively. The batch experimental results can be described by Freundlich equation, pseudo-second-order kinetics, and the intraparticle diffusion model, while the maximum adsorption capacity reached 239.6 mg g^−1^ under 318 K. The effects of flow rate, bed height, initial concentration, and temperature were studied in fixed bed adsorption experiments, and adsorption data were fitted with six dynamic adsorption models. The results of characterizations and the batch experiments were analyzed to study the adsorption and desorption mechanisms. Tetracycline hydrochloride and activated carbon were bonded together by π–π interactions and cation–π bonds. Ethanol was used as an eluent which bonded with 10 hydrogen bond acceptors on tetracycline hydrochloride to form a complex by hydrogen bonding to achieve recycling.

## 1. Introduction

Tetracycline (TCH) is the most widely used veterinary and human antibiotics type [1,2], however, approximately 50%–80% of the ingested TCH is excreted from the body through the urinary system [3]. TCH is a harmful residue if it is not been adequately pre-treated and is released into the soil or aquatic environment [4]. Generally, TCH with a low concentration (<1 mg L^−1^) in natural waters arouses great concerns of toxic effects and may have the potential to spread antibiotic resistant genes. It is an urgent task for us to remove the low concentrations of TCH for safe water supplies. The adsorptive method is a good solution, but it requires adsorbents with high adsorption capacity.

Activated carbon (AC) provides a stronger adsorption affinity to adsorbate among other adsorbents [5]. It is a low-cost and environmentally friendly choice, as a large number of abandoned bagasse resources are used to prepare activated carbon. Bagasse is not only rich in C atoms but also has numerous –OH groups, some of which remain in the pyrolysis, while other parts forms new chemically-reactive oxygen functional groups [6]. AC contains not only a benzene ring but also many chemically reactive oxygen functional groups (–OH, –COOH, etc.), which can be used for chemical modification [7]. The –NH_2_ group in the TCH can form a π–π interaction and a cation–π bond with the benzene ring on the ZnCl_2_ activated bagasse-based activated carbon (ZBAC). During the pyrolysis process, cellulose and hemicellulose in bagasse (about 50 wt% cellulose and 25 wt% hemicellulose) are decomposed at 202 °C to produce levoglucosan (LGA) and anhydrosugar polymers. The isomerization reaction occurs after LGA dehydration which forms other anhydrosugars and furans [8]. The pyrolysis product is polymerized to form activated carbon at 600 °C. The activated carbon produced by direct the pyrolysis of bagasse has a small specific surface area (S_BET_ was about 376.08 m^2^ g^−1^), and ZnCl_2_ helps to form more porous structures during pyrolysis (S_BET_ was about 831.23 m^2^ g^−1^) because ZnCl_2_ catalyzes the Scholl condensation reaction during pyrolysis. The above-mentioned macromolecular polycyclic compounds in bagasse form porous structures during pyrolysis at high temperatures [9].

However, how activated carbon’s effect on the fate of tetracycline hydrochloride (TCH) in water has become a question demanding a quick solution, and few reports have paid attention to the adsorption and desorption behavior and mechanism. Desorption behavior influences not only the fate of TCH in the environment, which is still a primary concern, but also the efficient recycling of the adsorbent [10]. The structure–activity relationship of adsorbents plays a significant component in the adsorption and desorption process. The adsorption affinity between the adsorbent and the adsorbate determines adsorption and desorption behavior. In this work, adsorption and desorption performances of tetracycline hydrochloride by activated carbon derived from sugar cane bagasse activated with ZnCl_2_ and their mechanisms were studied.

## 2. Results and Discussions

### 2.1. Materials Structure and Composition

**Figure 1:**
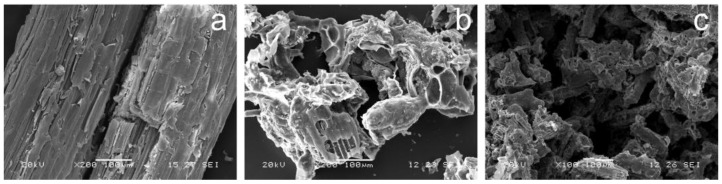
SEM images of (**a**) bagasse, (**b**) nCl2 activated bagasse based activated carbon (ZBAC), and (**c**) repeated use of ZBAC.

SEM images of bagasse, ZBAC and regenerated ZBAC ware shown in Figure 1. Figure 1b shows parallel tunnel-type pore structure compared to Figure 1a, which indicated that ZnCl_2_ activation and pyrolysis process contributed to the formation of a new pore structure in bagasse. This is one of the reasons why ZBAC had high-efficiency adsorption performance compared to BAC. It can be seen from Figure 1c that some of the pore structure remained after desorption, which indicated that ZBAC can be reused several times. What is more, S_BET_, D_P_, and V_mic_ of BAC and ZBAC were shown in Table S1, and ZBAC had a larger S_BET_, smaller D_P_, and larger V_mic_ than BAC. ZnCl_2_ catalyzed the Scholl condensation reaction and formed a porous structure during the carbonization process [9]. No sharp peak in ZBAC was shown in the XRD pattern (Figure S1a, marked with “AC”), which revealed a predominantly amorphous structure of activated carbon in ZBAC. The broad peak was an advantageous characteristic that made bagasse suitable for the preparation of the activated carbon adsorbent [11]. The TGA/DTGA of bagasse and ZIB are shown in Figure S2. There was no obvious weight loss above 600 °C in Figure S2b, therefore 600 °C was the minimum carbonization temperature for ZIB pyrolysis to prepare ZBAC [12]. FTIR spectroscopy of bagasse, ZBAC, and TCH load ZBAC are shown in Figure S1b. The bands at 1118, 1086, and 880 cm^−1^ were related to C–N stretching vibrations, C–H symmetric deformation vibrations, and N–H deformation vibrations [13]. Comparing the spectroscopy before and after adsorption, the band at 1086 cm^−1^ was weakened after adsorption and new bands of 880 cm^−1^ appeared. The ring structure in the TCH and aromatic rings in ZBAC promoted π–π interactions, and cation–π bonding occurred between the ZBAC and the easily protonated –NH_2_ which was located on the ring C4 of the TCH. FT-IR analysis confirmed the successful combination of ZBAC and TCH, indicating that bagasse was beneficial to TCH adsorption after ZnCl_2_ activation and pyrolysis [14].

### 2.2. Batch Adsorption Experiments

#### 2.2.1. Effect of pH

**Figure 2:**
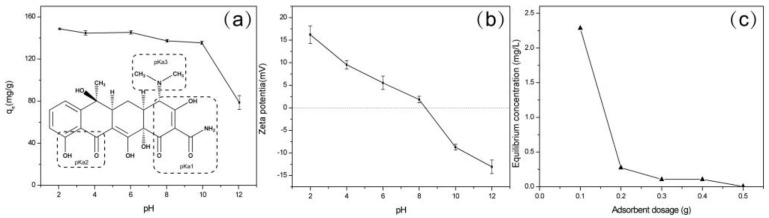
(**a**) Effect of pH (*C_0_* = 300 mg L^−1^, V = 50 mL, 0.1g ZBAC, T = 298 K); (**b**) Zeta potential of ZBAC as a function of pH; (**c**) Effect of adsorbent dosage (*C_0_* = 240 mg L^−1^, V = 100 mL, pH = 3.5, T = 298 K).

The pH of the TCH solution is an important parameter for controlling the adsorption capacity of the ZBAC. The adsorption of TCH decreased from 149.4–135.3 mg g^−1^ as the pH increased from 2 to 10 (Figure 2a). When the pH continued to increase to 12, the adsorption capacity slumped to 79.25 mg g^−1^. The adsorption efficiency was only 53% of the maximum when the pH was 12. These results indicated that many of the reactive functional groups sited on the surface of the ZBAC, such as –OH and –COOH, were passivated or blocked when pH > 10. This phenomenon usually occurred during the chemisorption. Tetracycline (TC) was an amphoteric substance with different ionizable groups on the molecule surface at different pH values (Figure 2a insert). The dominated TC (H_2_TC) species were H_3_TC^+^ at pH < 3.4, H_2_TC at pH of 3.4–7.6, HTC^−^ at pH of 7.6–9.0, and TC^2−^ at pH > 9 [13]. The pH_pzc_ of ZBAC was 8.3 (Figure 2b). Thus, the electrostatic attraction between TC and ZBAC increased with the decreasing of pH when pH < 8.3. Correspondingly, q_e_ of ZBAC decreased when pH > 8.3. This was mainly caused by the disadvantageous electrostatic conditions between TC (HTC^−^ and/or TC^2−^) and ZBAC surfaces [15].

#### 2.2.2. Adsorbent Dosage Studies

Different mass of ZBAC (0.1, 0.2, 0.3, 0.4, 0.5 g) was added in 240 mg L^−1^ TCH solutions, and the solution concentration at the equilibrium adsorption was measured (Figure 2c). The q_m_ was 119.8 mg g^−1^ when the ZBAC was 0.2 g. The adsorption performance of ZBAC was determined and the next experiment was designed through this experiment. Table 1 listed comparison of ZBAC adsorption capacity with several kinds of bioresource-based activated carbons.

#### 2.2.3. Kinetics Studies

**Figure 3:**
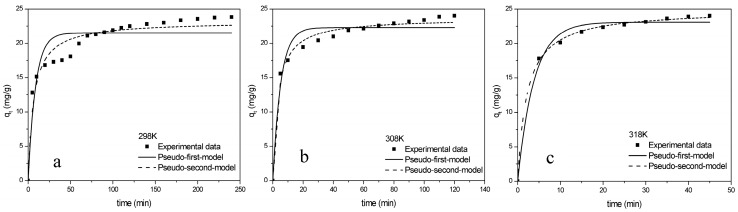
The Model I and Model II of TCH onto ZBAC (**a**) 298 K; (**b**) 308 K; (**c**) 318 K (*C_0_* = 120 mg L^−1^, pH = 6, V = 100 mL, ZBAC = 0.5 g).

The q_t_ was quickly reached near to the adsorption equilibrium in 30 min at 298 K (Figure 3). Then, it took a long time (120 min) to rise slowly and reach adsorption at the equilibrium point, while it took only 20 min at 318 K. Consequently, the time required to reach the adsorption equilibrium point was shorter as the temperature increased, which indicated that high temperature contributed to the adsorption process.

The non-linearized form of pseudo first-order kinetics model (Model I, Equation (1)) and the pseudo second-order kinetics model (Model II, Equation (2)) were used to simulate the kinetic data, as follows [20]:

(1) $$q_{t}\;=\;q_{e}(1-e^{-k_{1}t}),$$

(2) $$q_{t}\;=\;\frac{q_{e}^{2}k_{2}t}{{\;1\;+\;q}_{e}k_{2}t}$$

The initial adsorption rate equation (Equation (3)) [21]:

(3) $$k_{0}{\;=\;k}_{2}q_{e}^{2}$$

The adsorption kinetics fitted better with Model II than Model I (Table S2). The *R^2^* of Model II (0.9437, 0.9106, 0.9988) were higher than Model I (0.8523, 0.9481, 0.9864). Model II was believed to fit experimental data more accurately. Therefore, the adsorption behavior of TCH was followed by Model II, which indicated that the adsorption process was mainly via chemisorption [22].

TCH could permeate from the ZBAC surface to the inner surface by intraparticle diffusion. The intraparticle diffusion model (Model ID) was evaluated with the following Equation (4) [23]:

(4) $$q_{t}{\;=\;k}_{i}t^{\frac{1}{2}}\;+\;C$$

**Figure 4:**
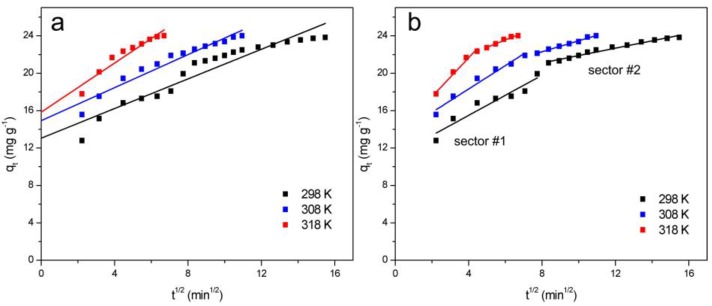
Intraparticle diffusion models of TCH onto ZBAC (**a**) overall fitting; (**b**) Two-sector fitting.

There were higher *R^2^* values (0.9083, 0.9426, and 0.9136) in the Model ID (Figure 4a) than Model I and Model II (Table S2). The results showed the intraparticle diffusion model could be better fitted with the adsorption process because of the porous structure of ZBAC, which was considered to be the key process of ZBAC adsorption TCH. In order to further clarify the rate-determining step and adsorption mechanism in the adsorption process, the Model ID was fitted into two sectors (Figure 4b), and the resulting parameters were shown in Table 2. In the first sector, TCH was diffused to the surface of ZBAC for adsorption in the liquid phase. In the second sector, TCH was gradually adsorbed on ZBAC, where intraparticle diffusion played a leading role in this sector. The slopes of the two sectors obtained through calculation were different. The slope of the first sector (1.073, 1.250, and 2.079) were much higher than the slope of the second sectors (0.3948, 0.5875, and 0.7908), indicating that the surface adsorption rate in the first sector was faster than the gradual adsorption rate in the second sector and intraparticle diffusion was not the only rate-determining step. The entire adsorption process was complex, which was determined by surface adsorption and intraparticle diffusion.

#### 2.2.4. Adsorption Isotherm

Three adsorption models were utilized to study the adsorption performance: Langmuir, Freundlich and Dubinin−Radushkevich isotherm models. Langmuir isotherm model (Model L) was a monolayer adsorption process with uniform distribution of adsorbates without any interaction [24]. The Langmuir isotherm was (Equation (5)) [23]:

(5) $$\frac{c_{e}}{q_{e}}\;=\;\frac{1}{q_{0}k}+\frac{c_{e}}{q_{0}}$$

The dimensionless constant separation factor (*R_L_*) could be used to judge whether the adsorption process was favorable for the Model L. The *R_L_* calculating equation was as follows (Equation (6)) [25]:

(6) $$R_{L}\;=\;\frac{1}{{1\;+\;\mathrm{Kc}}_{0}}$$

The value of *R_L_* was calculated to range from 0.001 to 0.022 (Table S3). As listed above, proving porous structure contributed to adsorption because the *R_L_* was not only in the range of 0–1 but also much less than 1.

While the Freundlich isotherm model (Model F) was multilayer adsorption, as followings (Equation (7)) [26]:

(7) $${\mathrm{lnq}}_{e}{\;=\;\mathrm{lnk}}_{f}\;+\;\frac{1}{n}{\mathrm{lnc}}_{e}$$

**Figure 5:**
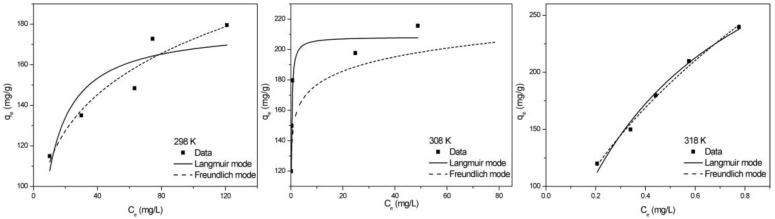
Adsorption equilibrium isotherm of TCH onto ZBAC: Model L and Model F fitting curve. (*C_0_* = 240, 300, 360, 420, 480 mg L^−1^, pH = 3.5, V = 50 mL, ZBAC = 0.1 g).

The value of 1/n was calculated to range from 0.007 to 0.538 (Table S4), which between 0–1, suggesting that TCH could be easily adsorbed on the ZBAC [27]. The adsorption isotherm curve and the fit curve were shown in Figure 5. Because the *R^2^* for TCH obtained from Model F (0.9048, 0.9070, and 0.9922) were higher than Model L (0.8011, 0.9499, and 0.9830), Model F could exactly match the experimental data in both models. The k_f_ (Freundlich constants) calculated from the Model F were 73.82, 148.9, and 276.7, which indicated that physisorption and chemisorption together constituted the adsorption process of ZBAC adsorption TCH [28].

Dubinin−Radushkevich isotherm model (Model DR) could be used to predict adsorption energy, as follows (Equation (8)) [29]:

(8) $${\mathrm{lnq}}_{e}{\;=\;\mathrm{lnq}}_{m}\;{-\;\varepsilon }^{2}$$

*K’* (*E*, kJ mol^−1^) was related to express the adsorption energy (Equation (9)):

(9) $$E\;=\;\frac{1}{{\left(2K^{\prime }\;\right)}^{\frac{1}{2}}}$$

Adsorption energy calculated from the figure of ln*q_e_* versus *E* were listed in Table S4. The adsorption process could be regarded as chemisorption when *E* > 8 kJ mol^−1^, which was in the range of 13.51~17.96 kJ mol^−1^ in this study. It was also found that the adsorption process had a high correlation with the Model ID (0.9068, 0.9961, and 0.9907). Therefore, the adsorption process in this study was closely related to chemisorption.

The type of adsorption can be decided by some thermodynamic parameters, such as Gibbs free energy (ΔG). ΔG is calculated as follows (Equation (10)):

(10) ΔG = − RTlnK

where K represents the adsorption equilibrium constant (from Langmuir model). The calculated ΔG under 298 K, 308 K, and 318 K were 1.827, 0.275, and –1.014 KJ mol^−1^ respectively. It can be seen that ΔG decreased with increasing temperature, from less than 0 to greater than 0, which indicated that the adsorption process of ZBAC was feasible and spontaneous thermodynamically when temperature increased. This was why initial rate of the adsorption capacity increase with increasing of the temperature.

### 2.3. Fixed Bed Adsorption Experiments

Define the following parameters according to the previous research: the breakthrough point is *C_t_*/*C_0_* = 10%, and saturation point is *C_t_*/*C_0_* = 90% [30]. These parameters of fixed bed adsorption were summarized in Table 3. The calculation methods for empty bed contact time (EBCT, Equation (11)) and mass transfer zone (MTZ, Equation (12)) were [30]:

(11) $$\mathrm{EBCT}\;=\;\frac{\mathrm{Bed}\;\mathrm{volume}}{\mathrm{Flow}\;\mathrm{rate}}$$

(12) $$\mathrm{MTZ}\;=\;H\left(1\;-\;\frac{t_{b}}{t_{s}}\right)$$

#### 2.3.1. Effect of Flow Rate

**Figure 6:**
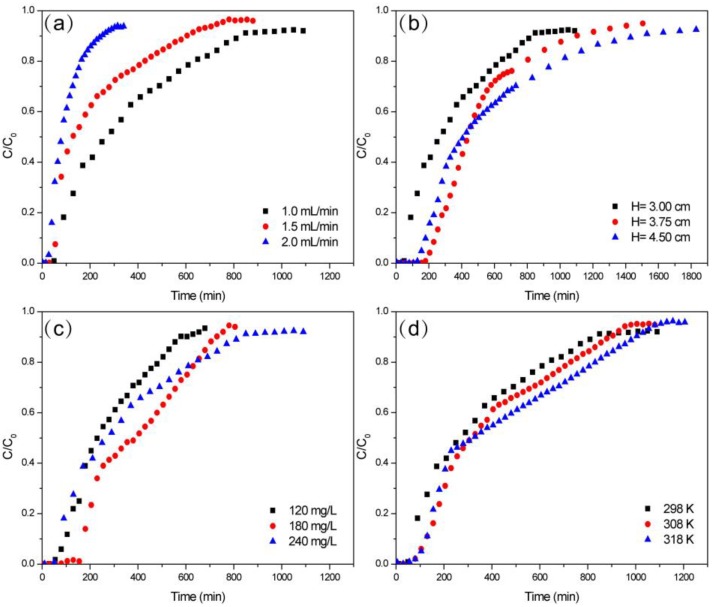
Breakthrough curves of TCH adsorption by ZBAC using fixed-bed columns of (**a**) different flow rates (C_0_ = 240 mg L^−1^, H = 3 cm, pH = 3.5, T = 298 K), (**b**) different bed height (Q = 1.0 mL min^−1^, C_0_ = 240 mg L^−1^, pH = 3.5, T = 298 K), (**c**) different initial concentrations (Q = 1.0 mL min^−1^, H = 3 cm, pH = 3.5, T = 298 K), and (**d**) different temperature (Q = 1.0 mL min^−1^, C_0_ = 240 mg L^−1^, H = 3 cm, pH = 3.5).

The experimental data under various *Q* were shown in Figure 6a. It had been observed the breakthrough curve had a steeper slope, and *t_b_* became shorter when *Q* increased. The slower *Q* provided a longer time for TCH to occupy the adsorption site on ZBAC. Accelerating flow rate contributed to the promotion of the mass transfer rate, while the adsorption capacity of ZBAC was not significantly reduced [18].

#### 2.3.2. Effect of Bed Height

The experimental data under various *H* were shown in Figure 6b. The increase in fixed bed height meant more mass of ZBAC and more longitudinal distribution. Diffusion of TCH into the ZBAC increases with the higher bed. It could conclude that *t_s_* increased significantly when *H* heightened. The higher *H* represented a longer EBCT, a wider mass transfer zone, and a lower slope during the adsorption process [31].

#### 2.3.3. Effect of Initial Concentration

The experimental data under various *C_0_* were shown in Figure 6c. It provided a stronger driving force when the *C_0_* of TCH increased [32]. Higher *C_0_* of TCH provided more TCH to bind to the adsorption site of ZBAC, which might be the reason for an increase in adsorption capacity. The adsorption procedure required more time to reach the equilibrium due to the *C_0_* of TCH increased, so the saturation time increased and the slope decreased.

#### 2.3.4. Effect of Temperature

The experimental data under various temperatures were shown in Figure 6d. It provided a stronger driving force and equilibrium concentration was closer to the initial concentration when the temperature rose. This result was consistent with the kinetic study that high temperatures accelerated the absorbed velocity and adsorption could reach equilibrium point quickly.

#### 2.3.5. Modelling of Breakthrough Curves

The calculated parameters of the six models at various experimental conditions are listed in Table 4. The equations for the six models are listed in Table S5 [30]. The *R^2^* of five of the models (except the Clark model) ranged from 0.9228 to 0.9966. The *R^2^* of the Clark model was calculated to fall between 0.8374 and 0.9246. This indicated well-fitting results between the experimental data and the fixed bed data generated from these models. Consequently, the Dose Response model was believed a fitting model for ZBAC adsorption of TCH because it had the highest *R^2^* amongst all models.

The q_0_ rose when *C_0_* increased in the Dose Response and Thomas model, which was consistent with the actual experimental data. Moreover, q_0_ rose as *H* increased in those two models, which may be attributed to an increase in the residence time of the TCH and an increase in the ZBAC mass. Similar results were found in the Adams–Bohart and BDST models, and as *C_0_* rose, volumetric adsorption capacity decreased [33]. *τ* in the Yoon–Nelson model indicated the time required to reach 50% retentions. *τ* reduced obviously as *Q* increases because the adsorption in the fixed bed reached saturation at a higher value of *Q* and the calculated value of *τ* was consistent with the experimental data.

### 2.4. Fixed Bed Desorption Experiments

**Figure 7:**
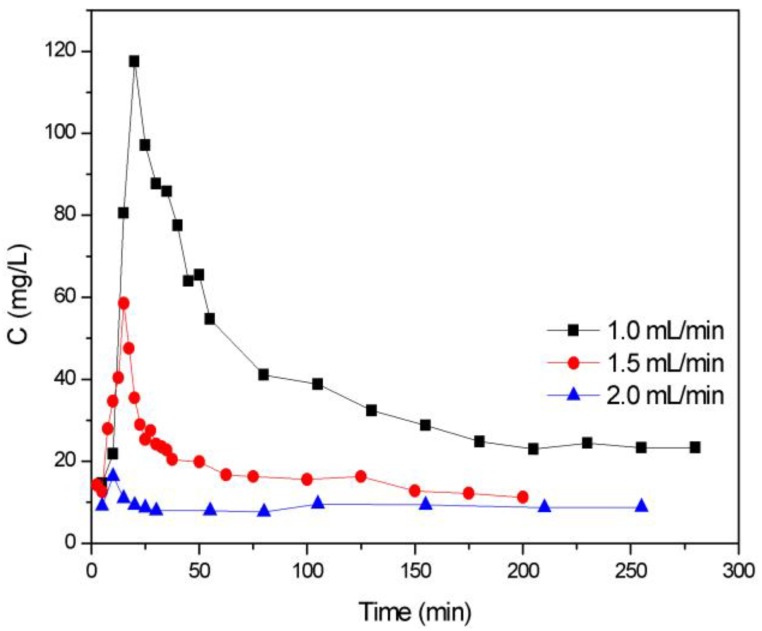
Elution curves for desorption of TCH from ZBAC by using anhydrous alcohol at different flow rates.

TCH (240 mg L^−1^) loaded ZBAC was desorbed by anhydrous alcohol which was regarded as eluent [34]. Ethanol provided hydrogen bonds that bound to hydrogen bond acceptors on the TCH for separating the TCH from the adsorbent active sites on the ZBAC. The elution curve was similar to the breakthrough curve, while referred to the desorption process. It was observed from Figure 7 that the elution curve had an asymmetrical distribution shape, and the released TCH concentration rapidly increased at the initial stage, followed by decreasing to a flat state.

Experimental breakthrough curves were used to calculate the amount of TCH desorbed per mass of ZBAC, as follows [35]:

(13) $$q_{\mathrm{total},d}\;=\;\frac{Q}{1000}\int_{t\;=\;0}^{{t\;=\;t}_{\mathrm{total},d}}C_{f}\mathrm{dt}$$

The amount of TCH desorbed, q_e,d_, through the following equation:

(14) $$q_{e,d}\;=\;\frac{q_{\mathrm{total},d}}{M}$$

Desorption parameters were summarized in Table 5. Although the maximum concentration (*C_p_*) could be reached quickly when the flow rate was increased, the total adsorption process concentration factor (*CF_p_* = *C_p_*/240) and desorption efficiency were decreased, which did not contribute to desorption [36]. It could be observed that the value of *CF_p_* was not high because the eluent was not completely effective and part of the TCH remained at the adsorbent site of the ZBAC. Consequently, this remained part of the TCH in desorption was considered to be irreversible adsorption. Comparing *C_p_* with 240 mg L^−1^, it was speculated that ZBAC can be recycled 2–3 times.

### 2.5. XPS Analysis

**Figure 8:**
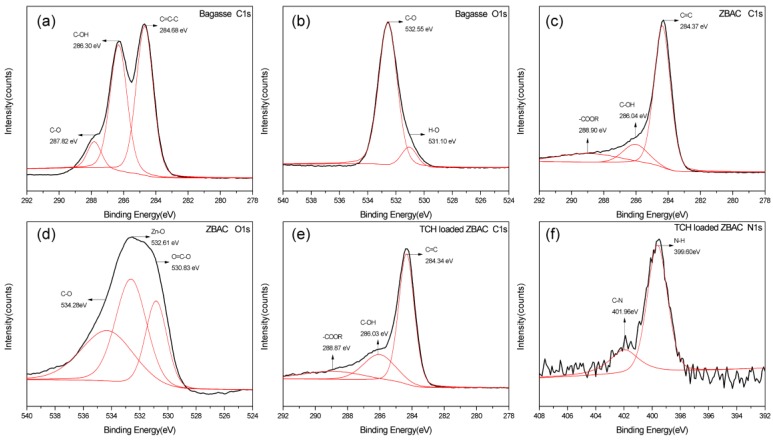
XPS spectra of bagasse and ZBAC: (**a**) C1s of bagasse, (**b**) O1s of bagasse, (**c**) C1s of ZBAC, (**d**) O1s of ZBAC, (**e**) C1s of TCH loaded ZBAC, and (**f**) N1s of TCH loaded ZBAC.

XPS analysis helps to further study the adsorption mechanism. Broadbands at 284.68, 286.30, and 287.82 eV in Figure 8a corresponded to C=C-C, C–OH, and C–O bonds in bagasse, respectively. Two bands at 531.10 and 532.55 eV in Figure 8b represented O–H and C–O in phenols, separately [37]. The C1s spectrum of ZBAC (Figure 8c) was resolved into three individual component bands: C=C (284.37 eV), C–OH (286.04 eV), and –COOR (288.90 eV). In the N1s of TCH loaded ZBAC spectrum, the bands at 399.60 and 401.96 eV correspond to N–H and C–N bonds, separately [38]. This also proved that TCH was successfully adsorbed onto the ZBAC. Comparing the C1s spectra of ZBAC- and TCH-loaded ZBAC, no difference was found in the functional group types, but the proportion slightly changed after adsorption. Lots of oxygen-containing groups were produced on the ZBAC after ZnCl_2_ activation and pyrolysis, such as –OH and –COOH, which might be involved in the adsorption of the TCH. The pH of the TCH in aqueous solution was 3.5, thus TCH existed in the cation form and acted as a π-electron-acceptor in the sorption process. In addition, some oxygen-containing groups on the ZBAC surface served as electron-donating groups [15]. Considering four aromatic rings in TC, the cation–π bonding might be one of the adsorption mechanisms [3]. In this study, cation–π bonding occurred between ZBAC and –NH_2_. The group of –NH_2_ which was located on the ring C4 of the TC could be easily protonated. The activated carbon was prepared by ZnCl_2_ activated bagasse and then pyrolysed, which provided many sites for adsorption, improving the adsorption capacity of TCH [15]. N–H on TCH bound to ethanol as a hydrogen bond acceptor after adsorption of TCH by ZBAC. Therefore, it could be speculated that hydrogen bonding of TCH and ethanol was the desorption mechanism of ZABC.

### 2.6. Adsorption and Desorption Mechanism

**Figure 9:**
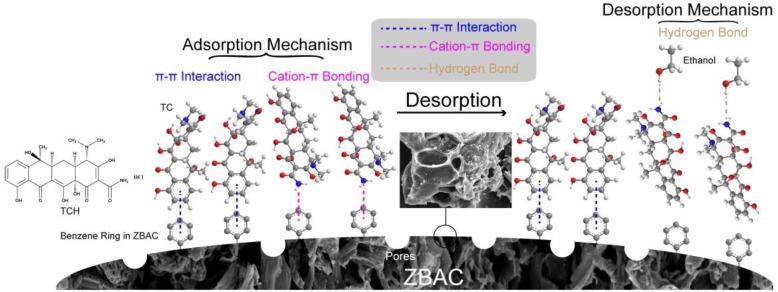
The schematic diagram illustrated the TCH adsorption and desorption mechanism on ZBAC.

There were two main adsorption mechanisms involved between ZBAC and TCH. They were intraparticle diffusion and chemisorption:

(**a**) The *R^2^* of Model ID were 0.9083, 0.9426, and 0.9136 when the temperature was in the range of 298–318K, respectively. The higher *R^2^* indicated that the TCH adsorption process on ZBAC fitted well with the intraparticle diffusion model, which was a vital process in the adsorption.

(**b**) The pH of TCH in aqueous solution was 3.5. The form of TCH was cation, acting as π-electron acceptor. The ZBAC surface had some oxygen-containing groups, such as –OH and –COOH, which served as electron-donating groups. It was speculated that ring structure in TCH and aromatic ring in ZBAC promote π–π interaction in them. Cation–π bonding occurred between the ZBAC and the easily protonated –NH_2_ which located on the ring C4 of the TCH. The adsorption mechanism was illustrated in Figure 9. There were two forms of existence of π–π interaction: Offset face-to-face and edge-to-face.

The desorption mechanism explained the procedure of elution of TCH on ZBAC by anhydrous ethanol. Ethanol was aprotic solvent that binds to 10 hydrogen bond acceptors in TCH, making TCH slightly soluble in ethanol. Figure 7 showed that a slower flow rate of TCH resulted in a higher desorption capacity, helping the TCH to separate from the ZBAC. However, the *CF_p_* value was low, indicating that the hydrogen bonding was insufficient to separate all of the TCH from the ZBAC. Therefore, this part of TCH that could not be desorbed was irreversible adsorption on ZBAC, which also indicated that ZBAC was relatively difficult to desorb while having higher adsorption capacity.

## 3. Materials and Methods

### 3.1. Materials

The bagasse was collected by Guangdong (China) Sugarcane Industry Research Institute. ZnCl_2_ (≥97%), HCl (37%), NaOH (≥96%), and anhydrous alcohol (≥99.5%) were purchased from the Chinese market. Tetracycline hydrochloride (USP) was purchased from Aladdin Industrial Corporation (Shanghai, China).

### 3.2. Preparation of the Absorbents

The preparation of the adsorbent in this study involved a two-step process: (1) impregnation of pre-carbonization bagasse with ZnCl_2_, followed by (2) pyrolysis with nitrogen at 600 °C (10 °C/min, 120 min) in a tube furnace (Carbolite, Modular Horizontal Tube Furnace, GHA 12/600) to develop the extended surface area and porous structure of ZBAC [39]. In the impregnation step, bagasse was pyrolyzed at 250 °C for 120 min at first, then the ZnCl_2_ was added to bagasse (impregnation ratio = 2:1, *w*/*w*), and finally, the mixture was desiccated at 100 °C for 24 h [40]. The sample of the impregnation step was called ZnCl_2_ impregnated bagasse (ZIB). The sample prepared without activation by ZnCl_2_ was named bagasse activated carbon (BAC).

### 3.3. Characterization

Morphological and structural analysis of bagasse and ZBAC surface was imaged using HitachiTM 3030(Hitachi, Ltd, Tokyo, Japan). Using Micromeritics ASAP 2020 (Micromeritics Instrument Corp, Norcross, USA) to determine the S_BET_, V_mic_, and D_P_ of the BAC and ZBAC. Crystallographic characterization was determined by XPert Pro (PANalytical, Malvern, WR14 1XZ, United Kingdom). Thermogravimetric experiments of bagasse and ZIB were tested using a thermogravimetric analyzer (HITACHI, STA7300, Hitachi, Ltd, Tokyo, Japan). FTIR analysis of bagasse and ZBAC were recorded using Nicolet 6700 spectrometer (Thermo Fisher Scientific, Waltham, MA USA 02451). Zeta potential analysis of ZBAC under diverse pHs (tested by PHS-3C, INESA Scientific Instrument Co., Ltd, Shanghai, China) was performed using Zetasizer Nano (Series Nano-ZS, Malvern, WR14 1XZ, United Kingdom). Surface chemical groups of the ZBAC and loaded ZBAC were analyzed by X-ray photoelectron spectroscopy (ESCALAB 250Xi, Thermo Fisher Scientific, Waltham, MA USA 02451).

### 3.4. Adsorption Experiments

ZBAC (0.1, 0.2, 0.3, 0.4, and 0.5 g) were put into TCH solution (*C_0_* = 120–240 mg L^−1^, V = 50 mL, pH = 3.5) in batch adsorption. The experimental parameters were described in detail in the figure caption. The mixed solution was shaken at different temperature (298, 303, and 313 K) for 48 h to ensure equilibrium adsorption was reached [41].

Fixed bed adsorption was performed in a glass column (φ = 12.5 mm, H = 60 mm). Different masses of ZBAC (0.8, 1.0, and 1.2 g) were added at the bottom of the glass column, filling the remaining part with quartz sand [30]. TCH concentrations were recorded at 274 nm by using UV-Visible spectrometer (SHIMADZU UV-1800, Kyoto, Japan) to obtain a breakthrough curve [42].

TCH (240 mg L^−1^) loaded ZBAC with the same mass was desorbed by anhydrous alcohol at three flow rates (1.0, 1.5, and 2.0 mL min^−1^), and a breakthrough curve was drawn accordingly.

## 4. Conclusions

Adsorption and desorption behaviors and mechanisms of bagasse-based activated carbon activated with ZnCl_2_ for TCH removal were studied by adsorption experiments, morphology characteristics and chemical properties analysis. The optimal ZBAC which had a maximum value of *S_BET_* as 831.23 m^2^ g^−1^, a *V_mic_* as 0.453 cm^3^ g^−1^, a *Dp* as 2.519 nm that were obtained by these conditions as follows: 2:1 impregnation ratio, 600 °C pyrolysis temperature for 120 min. The maximum adsorption capacity of ZBAC was 239.6 mg g^−1^ in this paper. The equilibrium data in batch adsorption experiments fitted well with Model II, Model ID, and Model F. Six mathematical models were used to fit the breakthrough curve in the fixed bed adsorption experiment, coming out that Dose Response model was the most fitted for the fixed bed model of ZBAC adsorption TCH. The elution curves were studied by using anhydrous alcohol. The slower flow rate was beneficial to increase *C_p_* and *CF_p_*, but desorption efficiency was lower overall. Some of the TCH remained at the adsorption site of ZBAC, which was considered to be irreversible adsorption. It was speculated that there were two adsorption mechanisms for TCH on ZBAC: a) Intraparticle diffusion on ZBAC, and b) π–π interaction and cation–π bonding between ZBAC and TCH. The desorption mechanism was that the hydrogen bond in the ethanol bound to the hydrogen bond receptor on the TCH, separating the TCH from the ZBAC. This study provides an important reference for a deeper understanding of the adsorption and desorption mechanisms of TCH on ZBAC. ZBAC as a recyclable renewable bio-resource material has potential in wastewater disposal.

## Figures and Tables

**Table 1:** Comparison of tetracycline hydrochloride (TCH) adsorption ZBAC ability with other bioresource-based activated carbon reported in references.

ACs	q_m_ (mg g^−1^)	References
Human hair	128.5	Ahmed, et al. [16]
Chicken feather	388.3	Li, Hu, Meng, Su and Wang [13]
Rice-husk	58.8	Chen, et al. [17]
Bamboo charcoal	27.7	Liao, et al. [18]
Rice husk ash	8.37	Liu, et al. [19]
Sugar cane bagasse	239.6	This study

**Table 2:** Model ID Parameters of TCH Removal onto ZBAC (two-sector).

Model ID	*q_e,exp_* mg g^−1^	*k_i_* mg^−1^ g min^−1/2^	*C* mg g^−1^	*R* ^2^
	Sector #1			
298 K	19.938	1.073	17.938	0.9013
308 K	21.885	1.250	13.310	0.9629
318 K	22.335	2.079	13.330	0.9696
	Sector #2			
298 K	23.818	0.3948	17.938	0.9743
308 K	23.999	0.5875	17.593	0.9891
318 K	23.999	0.7908	18.812	0.9571

**Table 3:** Breakthrough curve parameters for adsorption of TCH onto ZBAC.

No.	mL/min	cm	K	mg/L	pH	min	mL	mg/g	%	min	mL	mg/g	%	g	mg/L	min	cm
	Q	*H*	*T*	*C_0_*		*t_b_*	*V_b_*	q_b_	*R_b_*	*t_s_*	*V_s_*	q_s_	*R_s_*	*M*	*C_e_*	EBTC	MTZ
1	1	3	298	120	3.5	105	105	15.24	96.77	580	580	42.07	49.21	0.8	112.8	2.85	2.46
2	1	3	298	180	3.5	180	180	39.87	98.45	730	730	91.73	55.84	0.8	169.2	2.85	2.26
3	1	3	298	240	3.5	70	70	14.94	99.63	850	850	105.3	41.3	0.8	227.5	2.85	2.75
4	1	3.75	298	240	3.5	230	230	54.62	98.95	1105	1105	126.4	47.67	1	236.5	3.56	2.97
5	1	4.5	298	240	3.5	180	180	35.46	98.51	1530	1530	118.9	38.88	1.2	237.8	4.27	3.97
6	1.5	3	298	240	3.5	55	82.5	24.03	98.23	605	907.5	100.1	46.77	0.8	230.4	1.9	2.73
7	2	3	298	240	3.5	40	80	23.15	96.45	240	480	61.55	42.75	0.8	225.2	1.43	2.5
8	1	3	308	240	3.5	130	130	37.95	97.31	905	905	123.5	45.5	0.8	232.8	2.85	2.57
9	1	3	318	240	3.5	130	130	37.94	97.34	1005	1005	134.9	44.5	0.8	238.8	2.85	2.61

**Table 4:** Six models parameters at different conducted conditions.

Model	Parameters	1	2	3	4	5	6	7	8	9
Adams–Bohart	*K_AB_* (L mg^−1^ min^−1^) × 10^−5^	6.951	3.684	2.126	1.406	3.137	3.008	9.307	2.143	1.710
	*N_0_* (mg L^−1^) × 10^4^	0.932	2.041	2.367	2.293	2.469	2.154	1.300	2.692	2.984
	*R^2^*	0.958	0.955	0.9459	0.966	0.9659	0.937	0.945	0.945	0.928
Thomas	*K_th_* (L mg^−1^ min^−1^) × 10^−5^	6.967	3.685	2.071	1.969	3.137	3.002	9.309	2.164	1.694
	*q_0_* (mg g^−1^)	41.13	91.69	97.15	97.43	112.6	84.77	56.72	115.2	126.4
	*R^2^*	0.958	0.955	0.9412	0.923	0.9659	0.938	0.946	0.945	0.928
Yoon–Nelson	*K_YN_* (min^−1^) × 10^−2^	0.836	0.675	0.502	0.472	0.753	0.720	2.234	0.520	0.411
	*τ_cal_* (min)	274.1	403.5	320.6	487.1	469.4	188.4	94.53	384.2	417.0
	*R^2^*	0.958	0.955	0.9412	0.964	0.9659	0.938	0.945	0.945	0.928
BDST	*K_BDST_* (L mg^−1^ min^−1^) × 10^−5^	6.967	3.727	2.072	1.969	3.137	3.002	9.309	2.164	1.711
	*N_0_* (mg L^−1^) × 10^4^	0.894	1.974	2.112	2.118	2.449	1.843	0.616	25.06	2.720
	*R^2^*	0.958	0.955	0.9412	0.923	0.9659	0.937	0.945	0.945	0.928
Dose Response	*q_0_* (mg g^−1^)	36.68	78.25	82.15	89.09	108.9	66.66	49.28	98.66	103.4
	*α*	2.166	2.421	1.618	1.892	3.025	1.519	2.037	1.947	1.668
	*R^2^*	0.9940	0.974	0.9906	0.99	0.9934	0.98	0.997	0.988	0.968
Clark	A*10^3^	3.922	14.13	0.617	7.736	2.076	0.281	3.323	1.581	2.087
	*r* (min^−1^) × 10^−2^	1.764	1.492	0.981	1.152	1.869	1.302	4.995	1.036	0.956
	*R^2^*	0.902	0.918	0.8841	0.868	0.9246	0.837	0.89	0.884	0.882

**Table 5:** Desorption parameters for adsorption–desorption cycles, using a fixed-bed column for the removal of TCH (240 mg/L) by anhydrous alcohol-treated ZBAC (0.8 g) at different flow rate.

Q	q_total,d_	q_e,d_	t_p_	*C_p_*	*CF_p_*
mL/min	mg/g	mg/g	min	mg/L	
1.0	10.54	13.18	20	117.5	0.489
1.5	5.276	6.595	15	58.50	0.244
2.0	4.479	5.599	10	16.31	0.068

## Supplementary Materials

The following are available online, Figure S1: (**a**) XRD patterns of bagasse and ZBAC; (**b**) FT-IR spectra of bagasse, ZBAC and loaded ZBAC; Figure S2: TGA/DTG curves for bagasse and ZIB (**a**) bagasse, (**b**) ZIB. Tables S1: S_BET_, D_P_ and V_mic_ of BAC and ZBAC; Tables S2: Kinetic Parameters of TCH Removal onto ZBAC; Tables S3: *R_L_* values at various temperatures and initial concentrations; Tables S4: Constant parameter and correlation coefficients calculated for various adsorption models at different temperatures for TCH on ZBAC; Tables S5: Adams–Bohart, Thomas, Yoon–Nelson, BDST, Dose Response and Clark Model.

## Abbreviations

S_BET_	Brunauer–Emmett–Teller surface area (m^2^ g^−1^)
V_mic_	Micropore volume (cm^3^ g^−1^)
D_p_	Average pore diameter (nm)
q_m_	Maximum adsorption capacity of TCH per unit mass of ZBAC (mg g^−1^)
q_t_	Amounts of TCH adsorbed at contact time (mg g^−1^)
q_e_	Amounts of TCH adsorbed at equilibrium time (mg g^−1^)
k_1_	Rate constant for first order kinetic (min^−1^)
k_2_	Rate constant for second order kinetic (g mg^−1^ min^−1^)
k_0_	Initial adsorption rate (g mg^−1^ min^−1^)
K	Langmuir adsorption constant (L mg^−1^)
R_L_	Dimensionless constant separation factor
k_f_	Freundlich constant which indicates the adsorption capacity
n	Freundlich constant which related to the adsorption strength of the adsorbent
ε	Polanyi potential
K’	Constant of the adsorption energy (mol^2^ kJ^−2^)
K_i_	Rate constant of the intraparticle diffusion (mg g^−1^ min^−1/2^)
E	Adsorption energy (kJ mol^−1^)
pH_pzc_	pH at point of zero charge
H	Bed height in fixed bed column adsorption (cm)
Q	Flow rate (mL min^−1^)
t_b_	Breakthrough time (min)
V_b_	Volume of treated solution (mL)
q_b_	Adsorption capacity in Fixed bed adsorption (mg g^−1^)
R_b_	Metal removal efficiency of the breakthrough point (%)
t_s_	Saturation time (min)
V_s_	Volume of treated solution (mL)
q_s_	Adsorption capacity in Fixed bed adsorption (mg g^−1^)
R_s_	Metal removal efficiency of the saturation point (%)
M	Adsorbent dosage (g)
EBCT	Empty bed contact time (min)
MTZ	Mass transfer zone (cm)
C_f_	Desorbed concentration of TCH
q_e,d_	The amount of TCH desorbed
q_total,d_	Amount of TCH desorbed per mass of ZBAC
C_p_	The maximum concentration in desorption
CF_p_	The overall sorption process concentration factor

## References

[1] Ocampo-Pérez R., Rivera-Utrilla J., Gómez-Pacheco C., Sánchez-Polo M., López-Peñalver J.J. (2012). Kinetic study of tetracycline adsorption on sludge-derived adsorbents in aqueous phase. Chem. Eng. J..

[2] Parolo M.E., Savini M.C., Vallés J.M., Baschini M.T., Avena M.J. (2008). Tetracycline adsorption on montmorillonite: pH and ionic strength effects. Appl. Clay Sci..

[3] Gao Y., Li Y., Zhang L., Huang H., Hu J., Shah S.M., Su X. (2012). Adsorption and removal of tetracycline antibiotics from aqueous solution by graphene oxide. J. Colloid Interface Sci..

[4] Ji L.L., Chen W., Duan L., Zhu D.Q. (2009). Mechanisms for strong adsorption of tetracycline to carbon nanotubes: A comparative study using activated carbon and graphite as adsorbents. Environ. Sci. Technol..

[5] Zhang W., Lu Y., Sun H., Zhang Y., Zhou M., Song Q., Gao Y. (2019). Effects of multi−walled carbon nanotubes on pyrene adsorption and desorption in soils: The role of soil constituents. Chemosphere.

[6] Garg U.K., Kaur M.P., Sud D., Garg V.K. (2009). Removal of hexavalent chromium from aqueous solution by adsorption on treated sugarcane bagasse using response surface methodological approach. Desalination.

[7] Yu Z., Peldszus S., Huck P.M. (2009). Adsorption of selected pharmaceuticals and an endocrine disrupting compound by granular activated carbon. 2. Model prediction. Environ. Sci. Technol..

[8] Lin Y.C., Cho J., Tompsett G.A., Westmoreland P.R., Huber G.W. (2009). Kinetics and Mechanism of Cellulose Pyrolysis. J. Phys. Chem. C.

[9] Ahmadpour A., Do D.D. (1997). The preparation of activated carbon from macadamia nutshell by chemical activation. Carbon.

[10] Yubing S., Shubin Y., Yue C., Congcong D., Wencai C., Xiangke W. (2015). Adsorption and desorption of U(VI) on functionalized graphene oxides: a combined experimental and theoretical study. Environ. Sci. Technol..

[11] Kumar A., Mohan Jena H. (2015). High surface area microporous activated carbons prepared from Fox nut (Euryale ferox) shell by zinc chloride activation. Appl. Surf. Sci..

[12] Ozdemir I., Şahin M., Orhan R., Erdem M. (2014). Preparation and characterization of activated carbon from grape stalk by zinc chloride activation. Fuel Process. Technol..

[13] Li H., Hu J., Meng Y., Su J., Wang X. (2017). An investigation into the rapid removal of tetracycline using multilayered graphene-phase biochar derived from waste chicken feather. Sci. Total Environ..

[14] Chen A., Shang C., Shao J., Lin Y., Luo S., Zhang J., Huang H., Lei M., Zeng Q. (2017). Carbon disulfide-modified magnetic ion-imprinted chitosan-Fe(III): A novel adsorbent for simultaneous removal of tetracycline and cadmium. Carbohydr. Polym..

[15] Shan D., Deng S., Zhao T., Yu G., Winglee J., Wiesner M.R. (2016). Preparation of regenerable granular carbon nanotubes by a simple heating-filtration method for efficient removal of typical pharmaceuticals. Chem. Eng. J..

[16] Ahmed M.J., Islam M.A., Asif M., Hameed B.H. (2017). Human hair-derived high surface area porous carbon material for the adsorption isotherm and kinetics of tetracycline antibiotics. Bioresour. Technol..

[17] Chen Y., Wang F., Duan L., Yang H., Gao J. (2016). Tetracycline adsorption onto rice husk ash, an agricultural waste: Its kinetic and thermodynamic studies. J. Mol. Liq..

[18] Liao P., Zhan Z., Dai J., Wu X., Zhang W., Wang K., Yuan S. (2013). Adsorption of tetracycline and chloramphenicol in aqueous solutions by bamboo charcoal: A batch and fixed-bed column study. Chem. Eng. J..

[19] Liu P., Liu W.J., Jiang H., Chen J.J., Li W.W., Yu H.Q. (2012). Modification of bio-char derived from fast pyrolysis of biomass and its application in removal of tetracycline from aqueous solution. Bioresour. Technol..

[20] Luo X., Lei X., Cai N., Xie X., Xue Y., Yu F. (2016). Removal of Heavy Metal Ions from Water by Magnetic Cellulose-Based Beads with Embedded Chemically Modified Magnetite Nanoparticles and Activated Carbon. ACS Sustain. Chem. Eng..

[21] Ahmad Z.U., Yao L.G., Wang J., Gang D.D., Islam F., Lian Q.Y., Zappi M.E. (2019). Neodymium embedded ordered mesoporous carbon (OMC) for enhanced adsorption of sunset yellow: Characterizations, adsorption study and adsorption mechanism. Chem. Eng. J..

[22] Luo X., Lei X., Xie X., Yu B., Cai N., Yu F. (2016). Adsorptive removal of Lead from water by the effective and reusable magnetic cellulose nanocomposite beads entrapping activated bentonite. Carbohydr. Polym..

[23] Lei X., Dai X., Long S., Cai N., Ma Z., Luo X. (2017). Facile Design of Green Engineered Cellulose/Metal Hybrid Macrogels for Efficient Trace Phosphate Removal. Ind. Eng. Chem. Res..

[24] Naeem A., Westerhoff P., Mustafa S. (2007). Vanadium removal by metal (hydr)oxide adsorbents. Water Res..

[25] Luo X., Zeng J., Liu S., Zhang L. (2015). An effective and recyclable adsorbent for the removal of heavy metal ions from aqueous system: Magnetic chitosan/cellulose microspheres. Bioresour. Technol..

[26] Luo X., Liu C., Yuan J., Zhu X., Liu S. (2017). Interfacial Solid-Phase Chemical Modification with Mannich Reaction and Fe(III) Chelation for Designing Lignin-Based Spherical Nanoparticle Adsorbents for Highly Efficient Removal of Low Concentration Phosphate from Water. ACS Sustain. Chem. Eng..

[27] Kul A., Koyuncu H. (2010). Adsorption of Pb(II) Ions From Aqueous Solution by Native and Activated Bentonite: Kinetic, Equilibrium and Thermodynamic Study. J. Hazard. Mater..

[28] Luo X., Cai Y., Liu L., Zeng J. (2019). Cr(VI) adsorption performance and mechanism of an effective activated carbon prepared from bagasse with a one-step pyrolysis and ZnCl2 activation method. Cellulose.

[29] Cai Y., Liu C., Lu Y., Luo X., Zeng J. (2019). Bagasse-based adsorbent with nitric acid esterification and Fe(iii) chelation for the highly efficient removal of low concentration phosphate from water. New J. Chem..

[30] Luo X., Yuan J., Liu Y., Liu C., Zhu X., Dai X., Ma Z., Wang F. (2017). Improved Solid-Phase Synthesis of Phosphorylated Cellulose Microsphere Adsorbents for Highly Effective Pb2+ Removal from Water: Batch and Fixed-Bed Column Performance and Adsorption Mechanism. ACS Sustain. Chem. Eng..

[31] Wan Ngah W.S., Teong L.C., Toh R.H., Hanafiah M.A.K.M. (2012). Utilization of chitosan–zeolite composite in the removal of Cu(II) from aqueous solution: Adsorption, desorption and fixed bed column studies. Chem. Eng. J..

[32] Bulgariu D., Bulgariu L. (2013). Sorption of Pb(II) onto a mixture of algae waste biomass and anion exchanger resin in a packed-bed column. Bioresour. Technol..

[33] Calero M., Hernáinz F., Blázquez G., Tenorio G., Martínlara M.A. (2009). Study of Cr (III) biosorption in a fixed-bed column. J. Hazard. Mater..

[34] Aschermann G., Zietzschmann F., Jekel M. (2018). Influence of dissolved organic matter and activated carbon pore characteristics on organic micropollutant desorption. Water Res..

[35] Martín-Lara M.A., Blázquez G., Ronda A., Rodríguez I.L., Calero M. (2012). Multiple biosorption–desorption cycles in a fixed-bed column for Pb(II) removal by acid-treated olive stone. J. Ind. Eng. Chem..

[36] Lodeiro P., Herrero R., Sastre de Vicente M.E. (2006). Batch desorption studies and multiple sorption-regeneration cycles in a fixed-bed column for Cd(II) elimination by protonated Sargassum muticum. J. Hazard. Mater..

[37] Qu R., Feng M., Wang X., Qin L., Wang C., Wang Z., Wang L. (2014). Metal accumulation and oxidative stress biomarkers in liver of freshwater fish Carassius auratus following in vivo exposure to waterborne zinc under different pH values. Aquat. Toxicol..

[38] Barroso-Bogeat A., Alexandre-Franco M., Fernández-González C., Gómez-Serrano V. (2016). Activated carbon surface chemistry: Changes upon impregnation with Al(III), Fe(III) and Zn(II)-metal oxide catalyst precursors from NO3− aqueous solutions. Arab. J. Chem..

[39] Acharya J., Sahu J.N., Sahoo B.K., Mohanty C.R., Meikap B.C. (2009). Removal of chromium(VI) from wastewater by activated carbon developed from Tamarind wood activated with zinc chloride. Chem. Eng. J..

[40] Angin D. (2014). Production and characterization of activated carbon from sour cherry stones by zinc chloride. Fuel.

[41] Antón-Herrero R., García-Delgado C., Alonso-Izquierdo M., García-Rodríguez G., Cuevas J., Eymar E. (2018). Comparative adsorption of tetracyclines on biochars and stevensite: Looking for the most effective adsorbent. Appl. Clay Sci..

[42] Song Q., Fang Y., Liu Z., Li L., Wang Y., Liang J., Huang Y., Lin J., Hu L., Zhang J. (2017). The performance of porous hexagonal BN in high adsorption capacity towards antibiotics pollutants from aqueous solution. Chem. Eng. J..

