# Supplementary material for: Adsorption and Desorption Performance and Mechanism of Tetracycline Hydrochloride by Activated Carbon-Based Adsorbents Derived from Sugar Cane Bagasse Activated with ZnCl2
Source: Molecules. 2019 Dec 11;24(24):4534. doi: 10.3390/molecules24244534 (PMC6943745; doi:10.3390/molecules24244534)
Supplement: Supplementary file 1 [file molecules-24-04534-s001.pdf]

Supplementary Material

Adsorption and desorption performance and mechanism of tetracycline hydrochloride by activated carbon-based adsorbents derived from sugar cane bagasse activated with $ZnCl_2$

Yixin Cai [1, 2], Liming Liu[1, 2], Huafeng Tian[1, *], Zhennai Yang[1, *], Xiaogang Luo[1, 2, 3, *]

[1] Beijing Advanced Innovation Center for Food Nutrition and Human Health, Beijing Technology & Business University (BTBU), Beijing, 100048, China

[2] School of Chemical Engineering and Pharmacy, Wuhan Institute of Technology, LiuFang Campus, No.206, Guanggu 1st road, Donghu New & High Technology Development Zone, Wuhan 430205, Hubei Province, PR China

[3] School of Materials Science and Engineering, Zhengzhou University, No.100 Science Avenue, Zhengzhou City, 450001, Henan Province, PR China

---

* Corresponding author: Xiaogang Luo, Professor, Ph.D.
School of Chemical Engineering and Pharmacy, Wuhan Institute of Technology, 693 Xiongchu Avenue, Wuhan 430073, Hubei, China
Tel.: +86-139-86270668;
Email: xgluo0310@hotmail.com; xgluo@wit.edu.cn (X. Luo)
Corresponding author:Zhennai Yang, Professor, Ph.D.
School of Food and Chemical Engineering, Beijing Technology and Business University, Beijing 100048, China
E-mail: yangzhennai@th.btbu.edu.cn (Z. Yang).
Corresponding author: Huafeng Tian, Professor, Ph.D.
School of Material and Mechanical Engineering, Beijing Technology and Business University, Beijing 100048, China
E-mail address: tianhuafeng@th.btbu.edu.cn (H. Tian).

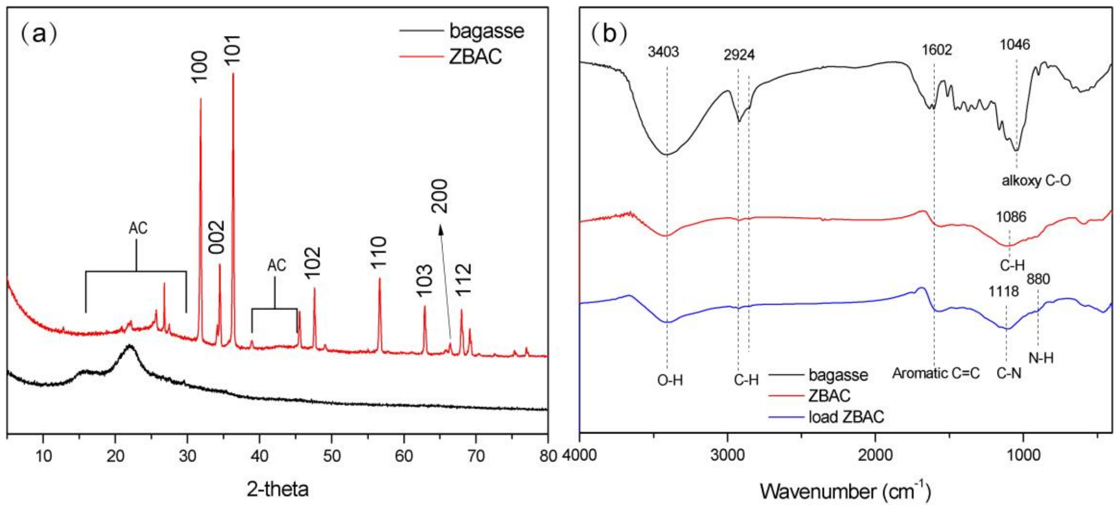

Figure S1. XRD patterns and FTIR of bagasse and ZBAC.

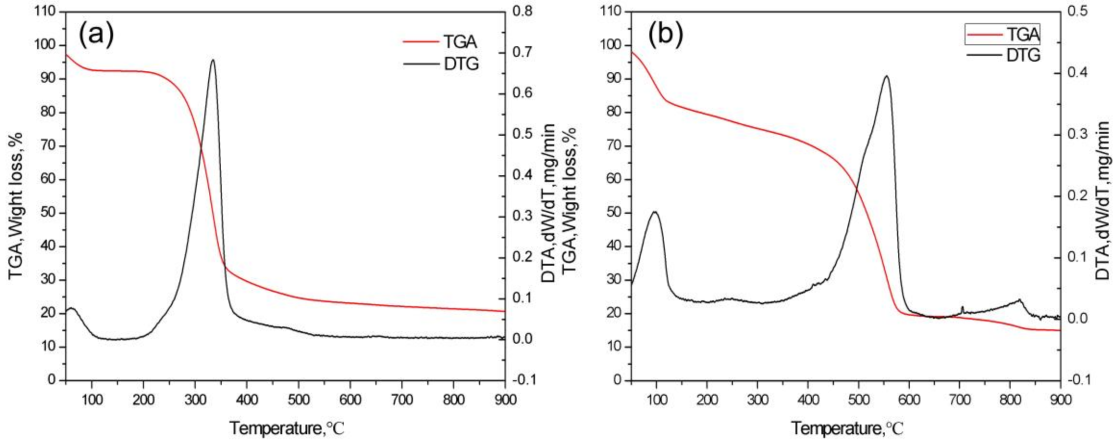

Figure S2. TGA/DTGA of bagasse and ZIB.

Table S1. S_{BET}, D_P and V_{mic} of BAC and ZBAC.

Samples	S_{BET} (m^2 g^{-1})	D_p (nm)	V_{mic} (cm^3 g^{-1})
BAC	376.08	2.826	0.186
ZBAC	831.23	2.519	0.453

Table S2. Kinetic Parameters of TCH Removal onto ZBAC.

Model Ⅰ	$q_{e,exp}$ mg g^{-1}	$q_{e,cal}$ mg g^{-1}	k_1 min^{-1}	R^2
298 K	23.818	21.505	0.116	0.8523
308 K	24.000	22.277	0.193	0.9481
318 K	24.000	23.070	0.262	0.9864

Model Ⅱ	$q_{e,exp}$ mg g^{-1}	$q_{e,cal}$ mg g^{-1}	k_2 g mg^{-1} min^{-1}	k_0 g mg^{-1} min^{-1}	R^2
298 K	23.818	22.232	0.011	5.310	0.9437
308 K	24.000	23.294	0.016	8.524	0.9106
318 K	24.000	24.765	0.020	12.137	0.9988

Model ID	$q_{e,exp}$ mg g^{-1}	k_i mg^{-1} g $min^{-1/2}$	C mg g^{-1}	R^2
298 K	23.818	0.792	13.059	0.9083
308 K	24.000	0.884	14.922	0.9426
318 K	24.000	1.311	15.846	0.9136

Table S3. R_L values at various temperatures and initial concentrations.

R_L	240 mg L^{-1}	300 mg L^{-1}	360 mg L^{-1}	420 mg L^{-1}	480 mg L^{-1}
298 K	0.022	0.018	0.015	0.013	0.011
308 K	0.005	0.004	0.004	0.003	0.003
318 K	0.002	0.001	0.001	0.001	0.001

Table S4. Constant parameter and correlation coefficients calculated for various adsorption models at different temperatures for TCH on ZBAC.

Isotherm equation		TCH		
		298 K	308 K	318 K
Langmuir	q_0 (mg g^{-1})	173.3	207.9	353.3
	K (L mg^{-1})	0.183	0.781	2.418
	R^2	0.8011	0.9499	0.9830
Freundlich	k_f	73.82	148.9	276.7
	1/n	0.184	0.007	0.538
	R^2	0.9048	0.9070	0.9922
DR	q_m (10^{-3} mol g^{-1})	0.707	2.381	15.13
	K' (10^{-2} mol^2 kJ^{-2})	1.550	2.010	2.741
	E (kJ mol^{-1})	17.96	15.77	13.51
	R^2	0.9068	0.9961	0.9907

Table S5. Adams−Bohart, Thomas, Yoon−Nelson, BDST, Dose Response and Clark Model.

Model	Equation	Parameters / Unit			
Adams-Borhart	$\frac{c_t}{c_0} = \frac{\exp(k_{AB}c_0t)}{\exp\left(\frac{k_{AB}N_0L}{v}\right) - 1 + \exp(k_{AB}c_0t)}$	k_{AB}	N_0	v	
		L mg^{-1} min^{-1}	mg L^{-1}	cm min^{-1}	
Thomas	$\frac{c_t}{c_0} = \frac{1}{1 + \exp[(\frac{k_{Th}}{Q})(q_0M - c_0Qt]}$	k_{Th}	q_0		
		mL min^{-1}	mg g^{-1}		
Yoon-Nelson	$\frac{c_t}{c_0} = \frac{\exp(k_{YN}t - \tau k_{NY})}{1 + \exp(k_{YN}t - \tau k_{NY})}$	K_{YN}	T		
		min^{-1}	min		
BDST	$\frac{c_t}{c_0} = \frac{1}{1 + \exp[k_{BDST}c_0(\frac{N_0}{c_0v} - t)]}$	k_{BDST}	N_0	v	L
		L mg^{-1}	mg L^{-1}	cm min^{-1}	cm
Dose Responded	$\frac{c_t}{c_0} = 1 - \frac{1}{1 + (\frac{c_0Qt}{q_0M})^a}$	α	q_0		
		-------------	mg g^{-1}		
Clark	$\frac{c_t}{c_0} = (\frac{1}{1 + Ae^{-rt}})^{\frac{1}{n-1}}$	A	r		
		-------------	Min^{-1}		
